# Unraveling the Genetic Foundations of Broiler Meat Quality: Advancements in Research and Their Impact

**DOI:** 10.3390/genes15060746

**Published:** 2024-06-06

**Authors:** Tian Lu, Bahareldin Ali Abdalla Gibril, Jiguo Xu, Xinwei Xiong

**Affiliations:** Jiangxi Provincial Key Laboratory of Poultry Genetic Improvement, Nanchang Normal University, Nanchang 330032, China

**Keywords:** chicken, meat quality, multiple factors, genomic approaches, molecular markers

## Abstract

As societal progress elevates living standards, the focus on meat consumption has shifted from quantity to quality. In broiler production, optimizing meat quality has become paramount, prompting efforts to refine various meat attributes. Recent advancements in sequencing technologies have revealed the genome’s complexity, surpassing previous conceptions. Through experimentation, numerous genetic elements have been linked to crucial meat quality traits in broiler chickens. This review synthesizes the current understanding of genetic determinants associated with meat quality attributes in broilers. Researchers have unveiled the pivotal insights detailed herein by employing diverse genomic methodologies such as QTL-based investigations, candidate gene studies, single-nucleotide polymorphism screening, genome-wide association studies, and RNA sequencing. These studies have identified numerous genes involved in broiler meat quality traits, including meat lightness (*COL1A2* and *ACAA2*), meat yellowness (*BCMO1* and *GDPD5*), fiber diameter (*myostatin* and *LncIRS1*), meat pH (*PRDX4*), tenderness (*CAPN1*), and intramuscular fat content (*miR-24-3p* and *ANXA6*). Consequently, a comprehensive exploration of these genetic elements is imperative to devise novel molecular markers and potential targets, promising to revolutionize strategies for enhancing broiler meat quality.

## 1. Introduction

Chicken muscle is one of the foremost sources of meat globally. It is characterized by its high protein content, nutritional richness, and versatile applications, making it a staple in many diets. Broiler meat quality refers to the characteristics obtained from broiler chickens specifically bred and raised for meat production. Chicken meat’s quality is a pivotal determinant of its palatability and sensory attributes. As consumer preferences increasingly veer towards high-quality food products, investigating chicken meat (broiler meat) quality traits garners escalating attention [1,2,3,4,5].

Critical chicken meat quality traits include meat color, water-holding capacity, pH levels, tenderness, and nutrient composition. These attributes influence the meat’s appearance, taste, shelf life, and nutritional profile. Consequently, a comprehensive understanding and exploration of chicken meat qualities are imperative to elevating poultry standards, meeting consumer expectations, and propelling advancements in the poultry industry.

In recent years, significant strides have been made in studying chicken meat quality traits [6,7,8,9]. Researchers have embraced various methodologies to assess, compare, and analyze chicken characteristics encompassing color, texture, pH, fat content, and nutritional composition. These endeavors have unveiled the underlying factors impacting these traits and elucidated strategies and methodologies for enhancing chicken meat quality [10,11].

However, knowledge gaps and challenges persist despite the progress in studying chicken meat quality traits. Notably, the full extent of different chicken hybrids and strains’ influence on meat quality traits remains elusive. At the same time, the effects of feed composition adjustments and breeding management strategies warrant further exploration. Therefore, this paper reviews and synthesizes the latest research advancements in chicken quality traits. Specifically, we will delve into meat color, water retention, pH levels, tenderness, and intramuscular fat. We will scrutinize the factors influencing these traits and elucidate policies and methodologies to enhance chicken meat quality. By providing comprehensive references and scientific insights, we aim to underpin the continual evolution of the chicken industry, offering guidance and inspiration for future research endeavors.

## 2. Genetic Factors Influencing Meat Quality Traits in Chicken

Genetic determinants play a pivotal role in shaping the qualitative attributes of chicken meat, encompassing characteristics such as flesh color, water retention, pH value, and tenderness. These genetic factors collectively contribute to chicken meat’s overall quality and sensory experience (Table 1).

### 2.1. Meat Color

Meat color is a vital visual indicator of quality for consumers, with brighter hues often associated with superior nutritional value and desirability [34]. Recent research has delved into the genetic underpinnings of meat color formation, unveiling candidate genes that influence flesh coloration. Studies have identified genes associated with glycolysis, fatty acid metabolism, protein metabolism, and heme concentration as potential regulators of meat color [7,12,14]. Notably, genes such as *ACAA2*, *ACSS3*, *APOH*, *ATP5L*, *CAV3*, *COL1A2*, *COX7C*, *GDPD5*, *MMP27*, *RBP4A*, *SLC2A6*, *TBXAS1*, and *UQCR10* have emerged as key players in modulating chicken meat color [6,7,8,12,13,15]. Understanding the genetic basis of meat color variation enables targeted breeding strategies and management practices to enhance meat quality and meet consumer preferences.

### 2.2. Water-Holding Capacity

Water-holding capacity (WHC) is a critical attribute affecting meat quality, as excessive water loss results in dry and unpalatable meat. Genetic studies have identified candidate genes in muscle cell membrane permeability, aquaporin protein regulation, and hydro-regulation pathways influencing WHC [35,36,37]. Notably, genes such as *ABCA1*, *COL6A1*, *GSTT1L*, and the IIb sodium phosphate cotransporter have regulated WHC and intramuscular fat deposition [35,36]. The IIb sodium phosphate cotransporter reduces the WHC of thigh meat [35].

The promoters of *ABCA1*, *COL6A1*, and *GSTT1L* were found to be hypermethylated, leading to transcriptional downregulation in hens during the later laying period. This downregulation may account for the significant difference in meat quality observed between juvenile and later-laying-period hens [36]. Woody breast muscle disease has also been associated with genetic mutations affecting WHC and overall meat quality [37]. Strategies aimed at modulating gene expression and optimizing muscle hydration levels can enhance WHC and improve the sensory attributes of chicken meat.

### 2.3. pH Value

The pH value of chicken meat plays a pivotal role in determining its flavor, texture, and overall quality. Genetic studies have identified genes involved in muscle pH regulation, including *ACOT9*, *APOO*, *CAV3*, *CUEO*, *E1BTD2*, *EIF2S3*, *KLH15*, *LOC107052650*, *MAPKAPK3*, *PCYT1B*, *PHKA1*, *PITX2*, *PPP1R3A*, *PRDX4*, *PRKAG2*, *RGCC*, *RHOC*, *SIX1*, *SLC25A30*, *SLC2A1*, *SLC37A4*, and *VTI1B*, which influence postmortem pH decline and ultimate meat pH [10,11,13,16,17]. Variations in these genes can impact muscle acidification rates and subsequent protein denaturation, affecting WHC and meat texture. Moreover, the temporal expression of genes such as *PITX2* and *SIX1* has been correlated with meat pH and sensory attributes, underscoring their role in meat quality determination [13].

### 2.4. Tenderness

Meat tenderness is a crucial factor influencing consumer perception and satisfaction. Genetic studies have identified candidate genes, including *CAPN1* and *CAST*, which regulate muscle protein degradation and influence tenderness [18,19]. Specific genetic variations within these genes, such as the SNP *CAPN1* G3535A and G37868A variant of the *CAST* gene, have been linked to variations in shear force values and intramuscular fat content [18,19]. Understanding the genetic mechanisms underlying meat tenderness enables targeted breeding programs and tenderization strategies to improve overall meat quality.

### 2.5. Muscle Fiber Diameter

Muscle fiber diameter is a crucial metric in assessing muscle structure and development, directly impacting the chewiness and texture of chicken meat [29,38]. Birds that grow rapidly have more muscle fibers than those that grow more slowly. Fast-growing chickens display larger muscle fiber diameters and a more significant proportion of glycolytic fibers (type II or quick-twitch, glycolytic fibers) than slow-growing chickens [39,40,41]. The fiber type distribution in different muscles influences their dominant metabolic capabilities, subsequently affecting the meat quality of slaughtered animals [42,43].

Understanding the genetic factors influencing muscle fiber diameter is paramount, prompting extensive research into associated genes. Identified candidate genes such as *myostatin*, *IGF-1*, and *PDK4* are implicated in various stages of muscle growth, fiber formation, and composition [28]. During chicken embryogenesis, *myostatin* downregulates *myf5*, *Pax3*, and *MyoD*, leading to a deficiency in limb muscle size [31]. Zhu et al. [28] employed qRT-PCR to measure the expression of these genes and utilized scanning electron microscopy for fiber diameter analysis. Their findings suggest that *myostatin*, *IGF-1*, and *PDK4* may significantly contribute to observed variations in chicken muscle characteristics.

Further investigations by Li et al. [27] highlighted the regulatory role of the hub gene *DYNLL2* and its target *miR-148-3p* in chicken myogenesis. *MyoD* and *MyoG*, members of the myogenic regulator family, activate transcription for myogenic agents by binding to a conserved E-box. They are crucial in muscle cell differentiation and development [30]. Interestingly, the long noncoding RNA *LncIRS1* has increased muscle fiber diameter and muscle mass in vivo in chickens [29]. Additionally, genetic strategies to modulate muscle hydration levels in broiler meat quality could include targeting *myomaker*, as elucidated by Luo et al. [30], which is essential for chicken myoblast fusion and subsequently impacts muscle development and possibly muscle hydration.

Moreover, the *IGFBP3* gene, integral to mammalian embryonic and postnatal development, inhibits chicken primary myoblast proliferation while promoting differentiation, thereby influencing muscle fiber diameter [8]. Boonlaos et al. [26] found that Toll-like receptor gene *TLR2* and *TLR4* mRNA expression correlate with the muscle fiber diameter of white striping chicken. Furthermore, the *IGF1* gene encoding Insulin-like Growth Factor 1 is crucial in muscle development [29,44]. Studies have revealed a correlation between *IGF1* gene polymorphism and chicken muscle fiber diameter, impacting meat texture and taste [44].

### 2.6. Crude Protein Content

Protein is a fundamental component of the muscles and tissues in chicken meat, directly influencing its nutritional value and overall quality. However, variations in protein composition among different chicken meat products lead to discrepancies in production and consumption. Consequently, researchers have explored genes associated with crude protein content in chicken meat to enhance its protein levels. Leveraging advancements in genomics and bioinformatics, researchers have identified candidate genes linked to crude protein synthesis and metabolism in chicken meat [32,33]. Bordini et al. [32] suggested that breast muscle width and total crude protein concentration can indicate enhanced breast yield and reduced aberrant composition in contemporary broilers. Hub genes within interconnected modules, such as *THRAP3*, *PRPF40A*, and *BIRC2*, play roles in oxidative stress adaptation and apoptosis inhibition, potentially influencing protein levels.

Moreover, genes encoding collagen variants (*COL5A2* and *COL6A3*) and *SPARC* and *MMP2* correlate strongly with crude protein content, indicating their significance in chicken meat production [32]. The *ATGL* gene, associated with chicken growth and fat traits, is strongly correlated with body weight and pectoral muscle crude protein content [33]. Similarly, studies suggest a relationship between *FABP* gene polymorphism and chicken meat crude protein content, potentially mediated through fatty acid–protein interactions [45]. Additionally, research indicates a correlation between *IGF1* gene polymorphism and chicken meat’s crude protein content, emphasizing its role in protein regulation [46].

Investigating the interplay between candidate genes and chicken meat’s crude protein content offers novel pathways for regulating protein synthesis and metabolism. However, current research on candidate genes in chicken meat remains nascent and requires further experimentation. Future studies, particularly utilizing gene editing techniques, can elucidate these genes’ functionality and regulatory mechanisms, contributing to enhanced chicken meat quality and increased protein content to meet the demand for high-quality meat [47].

### 2.7. Shear Force

The shear force of chicken meat, which represents the resistance encountered when cutting through the meat, is a crucial indicator for assessing the quality and tenderness of poultry meat. It is a significant parameter in determining meat quality, reflecting the strength and chewiness of muscle fibers. However, this force varies among different breeds and individuals, influenced by genetic factors. Hence, researchers have initiated studies on candidate genes associated with chicken meat’s shear force to unravel its genetic regulatory mechanisms [48].

Understanding the shear force of chicken meat can enhance food texture and cater to diverse consumer preferences. Piórkowska et al. [48] emphasized its importance in determining tenderness, a critical quality criterion. They identified genes like *ASB2*, associated with meat conversion by promoting protein breakdown, and genes involved in lipogenesis and collagen synthesis, potentially impacting shear forces. For instance, in regulating shear force, the *CAST* gene exhibited differential expression in lean and fat muscle [48]. Zhang et al. [49] highlighted its potential role in muscle fiber development and chicken shear force. Similarly, the *MSTN* gene encoding Mystatin, a muscle growth suppressor, showed associations with muscle growth and satellite cell proliferation, potentially influencing shear force [50].

Moreover, comprehensive gene expression analyses revealed essential genes like *PRKAG3*, *ATP2A2*, and *PPARGC1A* implicated in muscle fiber composition and transition [51]. *PPARGC1A*, in particular, emerged as crucial in muscle fiber dynamics in chickens. However, understanding these candidate genes’ functions and regulatory mechanisms is still in its infancy, necessitating further experimental validation. Future research employing gene editing and transgenic technology could unravel the mechanisms of these genes, optimizing the shear force and texture of chicken meat. Large-scale genome association studies and candidate gene screening hold promise in identifying additional genes related to chicken meat’s shear force, paving the way for enhancing meat quality and meeting consumer demands [47].

### 2.8. Intramuscular Fat Content

Intramuscular fat (IMF), nestled between muscle fibers, enhances the tenderness and flavor of chicken meat [52]. Research suggests that varying fat content indirectly influences meat quality characteristics such as tenderness, water content, and juiciness while significantly impacting flavor [53]. Optimal IMF levels maintain muscle wettability, ensuring meat softness and tenderness during cooking [53]. A higher fat content enhances juiciness and flavor richness [52]. Liu et al. [54] demonstrated that genetic selection for increased IMF strengthens fatty acid synthesis and transportation, improving chicken quality. Additionally, Lin et al. [22] revealed that *miR-24-3p* inhibits adipocyte differentiation, affecting intramuscular adipocyte deposition via *ANXA6* and, consequently, meat quality. *ANXA6* represses intramuscular preadipocyte proliferation while promoting differentiation [22]. Sun et al. [23] identified *miR-18b-3p*’s influence on IMF formation by targeting the *ACOT13* gene, potentially altering chicken meat quality characteristics. *miR-18b-3p* inhibits intramuscular preadipocyte differentiation, whereas *ACOT13* promotes differentiation. IMF deposition in breast muscle is associated with pyruvate and citric acid metabolism through *GAPDH*, *LDHA*, *GPX1*, and *GBE1* [21]. In Beijing-You and Cobb chickens, *ACADL*, *ACAD9*, *HADHA*, and *HADHB* were identified as candidate biomarkers for IMF deposition [24]. Cui et al. [55] explored the biochemical mechanisms underlying IMF deposition in chicken strains with increased fat content. Recently, a co-expression network analysis identified *miR-29c-3p*-*PIK3R1*, *miR-363-3p*-*PTEN*, *miR-6701-3p*-*PTEN*, *miR-449c/d-5p*-*TRAF6*, *miR-6701-3p*-*BMPR1B*, and *miR-1563*-*WWP1* as key miRNA and mRNA pairs controlling IMF accumulation [20] (Table 1).

The IMF content significantly influences chicken meat quality, with optimal levels enhancing tenderness, though excessive fat content can lead to greasiness. Therefore, controlling intramuscular fat content during breeding is crucial to ensuring favorable meat quality traits in chickens [55].

In conclusion, genetic factors are pivotal in determining meat quality traits in chickens, influencing characteristics such as flesh color, water-holding capacity, pH value, tenderness, muscle fiber diameter, crude protein content, shear force, and IMF content. By unraveling the genetic basis of these traits, researchers can develop targeted breeding strategies and management practices to enhance meat quality, meet consumer preferences, and drive advancements in the poultry industry.

## 3. Future Perspectives

Research into chicken meat quality traits has advanced significantly in recent decades, yet noteworthy challenges and opportunities warranting further exploration remain. Future investigations can delve into the foundational aspects of chicken meat quality traits, particularly by understanding their genetic underpinnings. By elucidating the genetic mechanisms governing these traits, breeders can enhance chicken meat’s quality and nutritional value through targeted breeding programs. Additionally, emphasis can be placed on refining feeding techniques and nutritional management practices, including optimizing feed composition, utilizing growth promoters, and enhancing feeding environments to maximize chicken meat quality.

The progression of science and technology opens up new avenues for studying chicken meat quality traits, with emerging technologies offering fresh opportunities. Modern biotechnological tools such as gene editing technology and metabolomics can be harnessed to analyze chicken meat quality traits, ultimately contributing to quality improvement. For instance, Tizard et al. [56] demonstrated the potential of gene editing and transgenic technology in controlling disease-related traits and removing allergens, showcasing the transformative impact of modern technologies in various production scenarios. Moreover, studies like that of Xu et al. [57] utilizing gene editing technology to elucidate the role of specific genes in chicken meat development further underscore the potential of these technologies.

However, integrating various analytical approaches can provide deeper insights into chicken meat quality development [20,58]. Research on chicken meat traits should encompass genetic exploration, muscle structure, physiological processes, feeding management, and advancements in nutritional technology. Such holistic investigations will be instrumental in elevating chicken meat quality to meet evolving consumer demands.

## 4. Summary

This article provides a comprehensive overview of the advancements in understanding the factors influencing chicken meat quality. The studies discussed have identified numerous genes associated with crucial broiler meat quality traits, such as meat lightness (*COL1A2* and *ACAA2*), meat yellowness (*BCMO1* and *GDPD5*), fiber diameter (*myostatin* and *LncIRS1*), meat pH (*PRDX4*), tenderness (*CAPN1*), and intramuscular fat content (*miR-24-3p* and *ANXA6*). These findings underscore the importance of investigating candidate genes to enhance chicken meat quality further and establish a solid foundation for future research.

## Figures and Tables

**Table 1:** Genes associated with meat quality attributes in chickens.

Attributes	Gene Symbol	Function	Study Model or Breeds	Technology Used for Identification	Refs.
Meat color	*ATP5L*, *UQCR10*, *COX7C*	Positively affects breast meat lightness and yellowness	Yellow-feather chickens (Huainan and Wannan chickens)	RNA-Seq/WGCNA	[8]
	*CAV3*, *RBP4A* and *APOH*	Negatively affects breast meat lightness and yellowness			
	*TBXAS1*	Regulates breast muscle redness	White-feathered chickens (Arbor Acres and Line B) and yellow-feathered chickens (Beijing-You and Jingxing Yellow) exhibit notable variations in meat color	mRNA sequencing/Selection signature analysis/qPCR	[7]
	*SLC2A6*, *MMP27* and *COL1A2*	Regulates breast muscle lightness			
	*GDPD5*	Positively affects breast muscle yellowness			
	*PHKG1*	Associated with breast muscle lightness	Ningdu yellow chickens	Association analysis of SNPs using DNA resequencing	[6]
	*ACAA2*	Regulates breast muscle lightness	Danzhou chickens	GWAS	[12]
	*ACSS3*	Regulates breast muscle redness			
	*PITX2*	CT genotype associated with highest breast muscle lightness	Wuliang Mountain Black-bone chickens	Association analysis of SNPs by DNA direct sequencing	[13]
	*COL1A2*	Associated with breast muscle lightness	F_2_ cross population between Beijing-You chickens and Cobb-Vantress	GWAS	[14]
	*BCMO1*	Associated with breast muscle yellowness	F_2_ cross population between high-growth and low-growth chicken lines	Expression QTL analysis	[15]
pH value	*SIX1*	GG genotype associated with the highest breast muscle pH	Wuliang Mountain Black-bone chickens	Association analysis of SNPs by DNA direct sequencing	[13]
	*PITX2*	CT genotype associated with highest breast muscle pH.			
	*PPP1R3A* and *SLC37A4*	Associated with muscle pH, potentially reduces pH	Two broiler lines with muscle high pH or low pH values	QTL and selection signature	[16]
	*MAPKAPK3*, *SLC25A30*, *RGCC*	Regulates breast muscle pH	Two broiler lines with muscle high pH (~6.34) or low pH (~5.55) values	Microarray	[11]
	*PRDX4*, *EIF2S3*, *PCYT1B*, *E1BTD2*	Associated with muscle pH, potentially reduces pH	F_2_ cross population between high-growth and low-growth chicken lines	Target enrichment analyses for a QTL and next-generation sequencing	[17]
	*PRDX4*	Associated with muscle pH and its expression downregulated	F_2_ cross population between high-growth and low-growth chicken lines	Expression QTL	[10]
	*ACOT9*				
	*KLH15*, *APOO*	Associated with muscle pH and its expression upregulated			
Tenderness	*CAPN1*	Associated with breast meat tenderness	Da-Heng Broiler	Association analysis of SNPs by DNA direct sequencing	[18]
	*CAPN1*	Associated with breast meat tenderness	Qingyuan partridge chicken and Recessive White chicken	Warner–Bratzler shear force/SNP by DNA direct sequencing	[19]
Intramuscular fat content	*miR-29c-3p*-*PIK3R1*, *miR-363-3p*-*PTEN*, *miR-6701-3p*-*PTEN*, *miR-449c/d-5p*-*TRAF6*, *miR-6701*-*3p*-*BMPR1B*, and *miR-1563*-*WWP1*	Regulates breast muscle IMF deposition	Beijing-You chicken	mRNA-Seq/small RNA-seq/WGCNA	[20]
	*LDHA*, *GPX1*, *GBE1*	Upregulated in high IMF	High and low IMF chickens from breast muscle	RNA-seq/network analysis	[21]
	*miR-24-3p*	Promotes intramuscular preadipocyte proliferation while inhibiting its differentiation by targeting *ANXA6*	In vitro	Overexpression and knockdown	[22]
	*ANXA6*	Inhibits intramuscular preadipocyte proliferation and promotes its differentiation	In vitro	Overexpression and knockdown	[22]
	*miR-18b-3p*	Inhibits intramuscular preadipocyte differentiation	In vitro	miRNA-Seq/miR-qPCR	[23]
	*ACOT13*	Enhances intramuscular preadipocyte differentiation	In vitro	miRNA-Seq/Luciferase assay/qPCR	[23]
	*ACADL*, *ACAD9*, *HADHA* and *HADHB*	Regulates IMF deposition. Upregulated before day 1 and downregulated from day 1 to day 14 after hatch	Beijing-You (slow-growing) and Cobb (fast-growing) chicken	Mass spectrometry-based approaches	[24]
Water Holding Capacity	*PHKG1*	Causes high drip loss	Ningdu yellow chickens	Association analysis of SNP by DNA direct sequencing	[6]
	*PRKAG3*	Associated with water-holding capacity	White Plymouth Rock	Association analysis of SNP by DNA direct sequencing	[25]
Muscle fiber diameter	*TLR2* and *TLR4*	mRNA expression correlates with muscle fiber diameter	Commercial poultry processing plant	Muscle cross-sectional area/qPCR	[26]
	*miR-148a-3p*	*miR-148a-3p* upregulates *MYHC* expression by targeting *DYNLL2*, promotes myoblast differentiation	In vitro	mRNA and miRNA analysis/overexpression/inhibition	[27]
	*DYNLL2*	Reduces MYHC expression, inhibits myoblast differentiation	In vitro	mRNA and miRNA analysis/overexpression/inhibition	[27]
	*Myostatin*, *IGF-1*, and *PDK4*	Expression correlates with muscle fiber diameter during embryogenesis	In vitro	Electron microscopy/qPCR	[28]
	*LncIRS1*	Increases muscle mass and mean muscle fiber	In vivo	Bioinformatic analysis/overexpression/shRNA/fiber cross-sectional area	[29]
	*miR-140-3p*	Inhibits myoblast fusion by targeting myomaker	In vitro	miRNA mimic and inhibitor/luciferase assay	[30]
	*Myomaker*	Promotes myoblast fusion	In vitro	Overexpression/siRNA	[30]
	*Myostatin*	*Myostatin* downregulates *Myf5*, *Pax3*, and *MyoD*, resulting in a deficiency in limb muscle size	In vivo	Overexpression/wholemount in situ hybridization	[31]
Crude protein content	*COL5A2*, *COL6A3*, *SPARC* and *MMP2*	mRNA levels covaried with crude protein content in normal and White Striping/Wooden Breasts	Ross 708 broiler	Microarray data collection/Co-expression network analysis; cytoHubba	[32]
	*ATGL*	Associated with crude protein content of breast muscle	F_2_ cross population between White Recessive Rock and Xinghua chickens	Association analysis of SNP by DNA direct sequencing	[33]

## Data Availability

No new data were created or analyzed in this study. Data sharing is not applicable to this article.

## Abbreviations

*ABCA1*—ATP-binding cassette sub-family A member 1, ACAA2—Acetyl-CoA acyltransferase 2, *ANXA6*—Annexin A6, *APOH*—Apolipoprotein H, *ATP5L*—ATP synthase membrane subunit L, *BCMO1*—Beta-carotene 15,15′-monooxygenase 1, *BIRC2*—Baculoviral IAP repeat-containing protein 2, *CAPN1*—Calpain-1 catalytic subunit, *CAV3*—Caveolin-3, *CAST*—Calpastatin, *COL1A2*—Collagen type I alpha 2 chain, *COL5A2*—Collagen type V alpha 2 chain, *COL6A*—Collagen type VI, *COL6A3*—Collagen type VI alpha 3 chain, *COX7C*—Cytochrome c oxidase subunit 7C, mitochondrial, *CUEO*—Copper-resistance protein CUEO, DEG—Differentially expressed in gastric cancer, *DYNLL2*—Dynein light chain LC8-type 2, *FABP*—Fatty acid-binding protein, *GDPD5*—Glycerophosphodiester phosphodiesterase domain-containing protein 5, *GSTT1L*—Glutathione S-transferase theta 1-like, *IGF-1*—Insulin-like growth factor 1, *IGFBP3*—Insulin-like growth factor-binding protein 3, *MMP2*—Matrix metalloproteinase-2, *MMP27*—Matrix metalloproteinase-27, *MyoD*—Myoblast determination protein 1, *PDK4*—Pyruvate dehydrogenase kinase 4, *PITX1*—Pituitary homeobox 1, *PITX2*—Pituitary homeobox 2, *PPP1R3A*—Protein phosphatase 1 regulatory subunit 3A, *PRDX4*—Peroxiredoxin-4, *PRKAG2*—Protein kinase AMP-activated non-catalytic subunit gamma 2, *PRPF40A*—Pre-mRNA-processing factor 40 homolog A, *PPKAG*—Protein kinase AMP-activated non-catalytic subunit gamma, *RBP4A1*—Retinol-binding protein 4, *SLC2A6*—Solute carrier family 2, facilitated glucose transporter member 6, *SIX1*—Homeobox protein SIX1, *SPARC*—Secreted protein acidic and rich in cysteine, *TBXAS1*—Thromboxane A synthase 1, *THRAP3*—Thyroid hormone receptor-associated protein 3, *TLR2*—Toll-like receptor 2, *TLR4*—Toll-like receptor 4, *TNNT3*—Troponin T3, fast skeletal type, *UQCP10*—Ubiquinol-cytochrome c reductase complex protein 10, WGCNA—Weighted gene co-expression network analysis.
